# Clinical and Radiologic Analysis of Minimally Invasive Anterior–Posterior Combined Surgery for Adult Spinal Deformity: Comparison of Oblique Lateral Interbody Fusion at L5/S1 (OLIF51) versus Transforaminal Interbody Fusion

**DOI:** 10.3390/medicina60010107

**Published:** 2024-01-06

**Authors:** Yoshihisa Kotani, Atsushi Ikeura, Takahiro Tanaka, Takanori Saito

**Affiliations:** 1 Spine and Nerve Center, Department of Orthopaedic Surgery, Kansai Medical University Medical Center, Moriguchi, Osaka 570-8507, Japan; aikeike55@mac.com (A.I.); takahiro.1124.t.t@gmail.com (T.T.); 2 Department of Orthopaedic Surgery, Kansai Medical University, Hirakata, Osaka 573-1191, Japan; saito@hirakata.kmu.ac.jp

**Keywords:** adult spinal deformity, minimally invasive surgery, oblique lateral interbody fusion, OLIF51, transforaminal interbody fusion

## Abstract

*Background and Objectives:* Although adult spinal deformity (ASD) surgery brought about improvement in the quality of life of patients, it is accompanied by high invasiveness and several complications. Specifically, mechanical complications of rod fracture, instrumentation failures, and pseudarthrosis are still unsolved issues. To better improve these problems, oblique lateral interbody fusion at L5/S1 (OLIF51) was introduced in 2015 at my institution. The objective of this study was to compare the clinical and radiologic outcomes of anterior–posterior combined surgery for ASD between the use of OLIF51 and transforaminal interbody fusion (TLIF) at L5/S1. *Materials and Methods:* A total of 117 ASD patients received anterior–posterior correction surgeries either with the use of OLIF51 (35 patients) or L5/S1 TLIF (82 patients). In both groups, L1–5 OLIF and minimally invasive posterior procedures of hybrid or circumferential MIS were employed. The sagittal and coronal spinal alignment and spino-pelvic parameters were recorded preoperatively and at follow-up. The quality-of-life parameters and visual analogue scale were evaluated, as well as surgical complications at follow-up. *Results:* The average follow-up period was thirty months (13–84). The number of average fused segments was eight (4–12). The operation time and estimated blood loss were significantly lower in OLIF51 than in TLIF. The PI-LL mismatch, LLL, L5/S1 segmental lordosis, and L5 coronal tilt were significantly better in OLIF51 than TLIF. The complication rate was statistically equivalent between the two groups. *Conclusions:* The introduction of OLIF51 for adult spine deformity surgery led to a decrease in operation time and estimated blood loss, as well as improvement in sagittal and coronal correction compared to TLIF. The circumferential MIS correction and fusion with OLIF51 serve as an effective surgical modality which can be applied to many cases of adult spinal deformity.

## 1. Introduction

With the increasing aging population, patients with adult spinal deformity (ASD) have been frequently treated conservatively or surgically. Clinical symptoms of ASD include intractable low back pain, impaired standing and walking ability, trunk or gazing disturbance, shortness of breath, eating disorders or gastroesophageal reflux, and psychological stress [1]. Neurologic disturbances also present as pain in lower extremities and intermittent claudication.

The quality of life of ASD patients is severely impaired compared to other chronic medical disorders [2,3]. Pellise et al. compared the physical and mental SF-36 scores between ASD and other chronic disorders from a database of 24,946 people, demonstrating lower physical and mental scores for ASD than those for disorders of diabetes, chronic lung disease, and congestive heart failure [2]. Bess et al. also reported lower physical SF-36 scores for symptomatic ASD compared to those for depression, diabetes, and hypertension within the total population of the United States [3]. Further impact was demonstrated whereby the physical SF-36 score for ASD was equivalent to that reported by cancer patients.

For severely disabled patients, surgical correction and fusion surgeries are usually performed. The effectiveness of surgical treatment has been discussed and evaluated, considering whether the surgical treatment improved patients’ quality of life or not [4,5]. Paulus et al. conducted a systematic review of thirteen studies evaluating the cost and value of spinal deformity surgery [4]. Using the measures of Scoliosis Research Society (SRS)-22 and Oswestry Disability Index (ODI), they concluded that ASD patients benefited from surgical treatment compared to nonsurgical treatment. Nonsurgical treatment did not seem to be cost-effective and did not have a positive impact on the quality of life of ASD patients. Acaroglu et al. evaluated a database of 968 ASD patients and compared the effectiveness of surgery between surgical and nonsurgical groups [5]. They demonstrated a superior effectiveness of 54% in the surgical group compared to 9.7% in the nonsurgical group.

Although surgery improves the quality of life of ASD patients, corrective ASD surgeries are accompanied by several complications [6,7]. Smith et al. reported a total of 70% complication rates in posterior-based ASD surgeries, with 52% at the perioperative and 43% at the delayed phases [6]. Sciubba et al. investigated 11,692 ASD patients and reported 55% overall complication rate [7]. They demonstrated that a higher complication rate was detected in three-column osteotomy surgeries.

Within the recent decade, several innovative surgical modalities have been developed to minimize the surgical invasiveness of ASD surgeries. Hynes et al. developed the oblique lateral interbody fusion (OLIF) technique, minimizing the conventional anterior lumbar interbody fusion (ALIF) surgery using a special tubular retractor (Medtronics, Memphis, TN, USA) [8]. The percutaneous pedicle screw (PPS) technique was successfully developed by Foley and the deformity correction with PPS was introduced by Anand et al. [9,10]. We started employing OLIF surgery for L1–5 in 2012 with combined use of PPS for ASD surgeries. Although successful clinical results with minimized invasiveness were obtained, there were still mechanical complications of rod fracture, proximal and distal junctional kyphosis, and pseudarthrosis seen in patients who received the surgery. To better solve these problems, L5/S1 OLIF (OLIF51) has been employed since 2015 instead of L5/S1 transforaminal interbody fusion (TLIF) [11,12].

The objective of this study was to compare the clinical and radiologic results of anterior and posterior combined correction surgery for ASD between the use of OLIF51 and L5/S1 TLIF.

## 2. Materials and Methods

**Figure 1:**
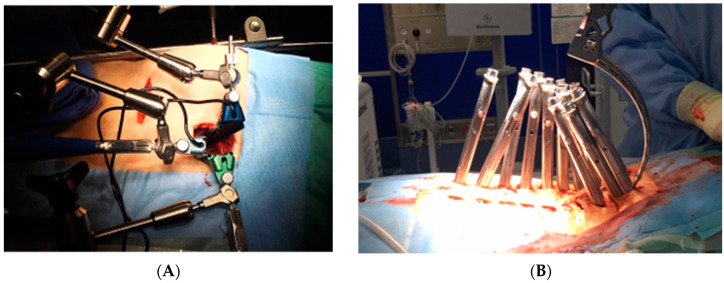
Circumferential MIS procedure with multiple OLIFs and all percutaneous posterior corrections. (**A**) The OLIF51 procedure utilizes the specially designed triple-arm retractor. (**B**) All percutaneous posterior correction procedures.

**Figure 2:**
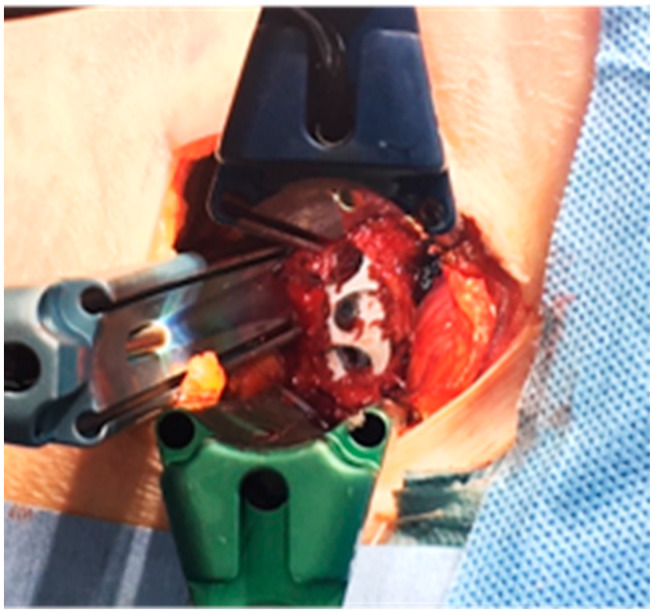
OLIF51 with use of the Sovereign hyperlordotic cage fixed with integrated screws and demineralized bone matrix soaked with aspirated bone marrow.

A total of 117 patients treated via anterior–posterior combined surgery with OLIF25 were enrolled in this study. The average age at the time of surgery was seventy-six years (54–86). In all patients, a two-stage surgery of multiple OLIFs followed by all percutaneous pedicle screw (PPS) correction (circumferential MIS) or combination of PPS and mini-open posterior correction was performed (Figure 1). Eighty-two patients received TLIF at L5/S1 (51TLIF group) and thirty-five patients received OLIF at L5/S1 (OLIF51 group). In the 51TLIF group, OLIF at L1–5 surgery was conducted at the first stage, and second-stage surgery included mini-open TLIF at L5/S1 using local bone and an 18-degree lordotic cage (Capstone control, Medtronics, Memphis, TN, USA) and correction with PPSs from the lower thoracic region to the pelvis. The OLIF51 group received L1-S1 OLIFs during the first stage, followed by PPS correction and fixation from the lower thoracic region to the pelvis (Circumferential MIS: cMIS). During the OLIF51 surgery, a 35 mm oblique incision was made, two-finger medially from the anterior superior iliac spine. Following the dissection of abdominal muscles, the retroperitoneal space was enlarged and bilateral common iliac vessels retracted. The placement of a triple-arm retractor safely secured the anterior aspect of the L5/S1 disc followed by discectomy and cartilaginous endplate removal. A 10-degree lordotic polyetheretherketone (PEEK) cage (MectaLIF, Medacta international, San Pietro, Switzerland), 12- or 18-degree lordotic PEEK cage (Sovereign, Medtronics, Memphis, TN, USA) with a demineralized bone matrix (DBM) soaked with aspirated bone marrow was placed in the disc space (Figure 2). The Sovereign cage allowed for the use of integrated screws for enhancing the fixation. For OLIF25, a rectangular PEEK cage (Clydesdale, Medtronics, Memphis, TN, USA) with demineralized bone matrix (DBM) soaked with aspirated bone marrow was placed at each disc level. In the posterior part of the cMIS surgery, long cortical bone trajectory screws were utilized for all PPSs, and two sacral alar–iliac screws (SAI) were used for pelvic fixation. The rod bent with lumbar lordosis and thoracic kyphosis was passed percutaneously from the caudal end to the cephalad end of the screw. Using the reducers of percutaneous screws, the rod was reduced to the screw head and fixed from the cephalad to the SAI, thereby completing the correction of the deformity. The MEP combined with SEP was monitored during all posterior parts of surgery. All surgeries were conducted with use of surgical navigation and an O-arm (Stealthstation 7, Medtronics, Memphis, TN, USA). 

The evaluated parameters comprised the total operation time (min, OT), estimated blood loss (mL, EBL), and early and late complications. The following radiologic parameters were measured preoperatively and at follow-up by two independent spine surgeons: sagittal vertebral axis (SVA; mm), coronal vertebral axis (CVA; mm), lumbar lordosis (LL; deg), lower lumbar lordosis (LLL; mm), sacral slope (SS; deg), pelvic incidence–lumbar lordosis mismatch (PI-LL; deg), pelvic tilt (PT; deg), segmental lordosis at L5/S1, and coronal tilt of the L5 vertebra. The clinical outcomes were evaluated via the Japanese Orthopaedic Association Back Pain Evaluation Questionnaire (JOABPEQ) effectiveness rate [13] and a visual analogue scale (VAS) preoperatively and at follow-up. The JOABPEQ questionnaire consisted of five domains: pain, low back function, gait, social and psychological parameters; a high effectiveness rate (%) indicates a better outcome. The patient demographics, shown in Table 1, demonstrated no significant differences between the two groups. 

Statistical analysis was conducted using EZR software (R version 4.2.2, The R foundation for statistical computing 2022). The *t*-test was performed between the two groups with a significance set at *p* = 0.05. This study was conducted based on the acceptance of the university ethics committee, and patient data and radiographs without personal information in this report are shown with the consent of the patients.

## 3. Results

**Figure 3:**
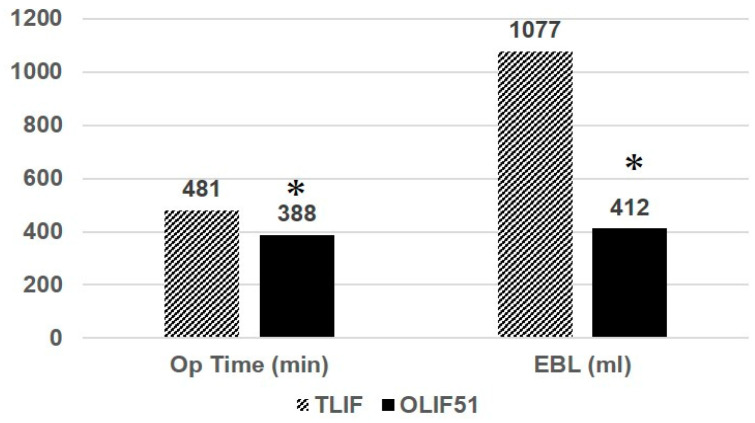
Operation time and estimated blood loss (EBL) for the two groups. The asterisk depicts statistical significance at the *p* < 0.05 level between two groups.

The average follow-up periods were 41 months (16–84) for the 51TLIF group and 26 months (13–42) for the OLIF51 group. The average number of fixed vertebrae was 8.1 (4–12) for the 51TLIF group, and 8 (4–10) for the OLIF51 group. The average OT was 481 min (278–739) and 388 min (267–608) for the 51TLIF and OLIF51 groups, respectively (*p* < 0.05) (Figure 3). The average EBL was 1077.9 mL (220–2644) and 411.7 mL (120–1708) for the 51TLIF and OLIF51 groups, respectively (*p* < 0.01). The coronal cobb angle was 25.1 degrees (4–45) and 29.0 degrees (2–72) for the 51TLIF and OLIF51 groups, preoperatively, and corrected to 9.5 degrees (1–23) and 10.0 degrees (1–26), respectively, at follow-up (NS) (Table 2). The thoracic kyphosis was 20.9 degrees (1–78) and 23.8 degrees (2–73) for the 51TLIF and OLIF51 groups, preoperatively, and corrected to 36 degrees (13–58) and 38.3 degrees (18–59), respectively, at follow-up (NS). The average CVA was 51.2 mm (19–123) and 33.9 mm (0–112) for the 51TLIF and OLIF51 groups, preoperatively, and corrected to 29.2 mm (0–53) and 17.0 mm (0–58), respectively, at follow-up (NS). The average SVA was 148 mm (10–349) and 149 mm (0–691) for the 51TLIF and OLIF51 groups, preoperatively, and corrected to 33.7 mm (−33.1–112) and 26.9 mm (−35–99), respectively, at follow-up (NS). The average SS was 21.4 deg. (11.6–33.4) and 17.0 deg. (−28–44) for the 51TLIF and OLIF51 groups, preoperatively, and corrected to 28.3 deg. (15.0–35.4) and 30.0 deg. (11.0–50.5), respectively, at follow-up (NS). The average PT was 30.9 deg. (18.3–48.1) and 31.2 deg. (14.0–54.0) for the 51TLIF and OLIF51, preoperatively and corrected to 16.7 deg. (4.9–36.0) and 19.0 deg. (−3.0–35), respectively, at follow-up (NS). 

The average lumbar lordosis (LL) was 7.6 deg. (−29.3–37.3) and 9.5 deg. (−38.0–50.1) for 51TLIF and OLIF51, preoperatively and corrected to 45.4 deg. (33.2–58.9) and 46.9 deg. (22.0–65.0), respectively, at follow-up (NS). The average PI-LL was 44.6 deg. (4.3–91.5) and 39.4 deg. (0–77.6) for 51TLIF and OLIF51, respectively, and corrected to 6.9 deg (−9.6–33.6) and 2.5 deg. (−23.7–24.0) at follow-up (*p* < 0.05). The average LLL was 18.1 deg. (−10.4–34.8) and 20.0 deg. (−26.7–47.2) for 51TLIF and OLIF51, preoperatively and corrected to 28.8 deg. (21.0–34.0) and 34.9 deg. (13–51.7), respectively, at follow-up (*p* < 0.01). The average lordotic angle at L5/S1 was 10.5 deg. (−1.1–22.2) and 11.3 deg. (−3–29.7) for 51TLIF and OLIF51, respectively, and corrected to 16.0 deg. (4.5–21.0) and 19.8 (5.6–44.1) at follow-up (*p* < 0.01). The average coronal tilt angle of L5 to S1 was 5.4 deg (0.6–14.5) and 6.0 deg (0–23) for 51TLIF and OLIF51, preoperatively and corrected to 4.3 deg. (0–10.5) and 2.5 deg. (0–8.0), respectively, at follow-up (*p* < 0.01).

**Figure 4:**
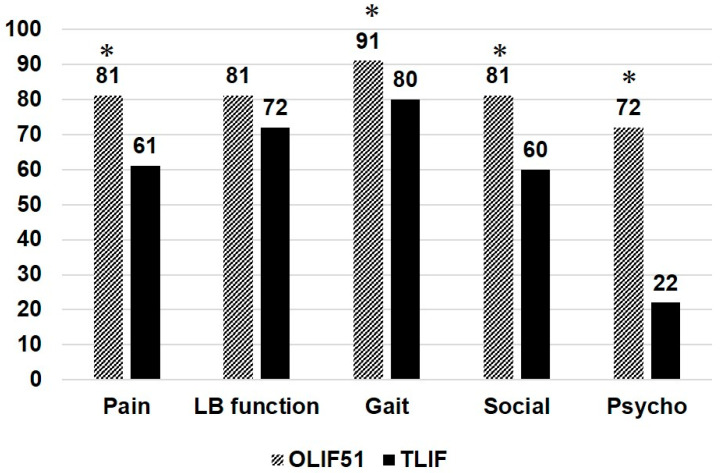
JOABPEQ effectiveness rate (%) for the two groups. The asterisk depicts statistical significance at the *p* < 0.05 level between two groups.

Figure 4 shows the JOABPEQ effectiveness rate (%) for the 51TLIF and OLIF51 groups, demonstrating several significant differences between the two groups. The effectiveness rate for OLIF51 was higher in pain, gait, social, and psychologic domains compared to the 51TLIF group (*p* < 0.05). Table 3 shows the VAS changes in the two groups between the preoperative stage and at follow-up. Both groups demonstrated significant reductions in VAS values after surgery; however, there were no statistical differences between the two groups. 

Remarks: The average number and range are depicted in each category. There was no statistical difference for all categories between OLIF51 and MIS-TLIF. LBP: low back pain, LE: lower extremity.

In terms of surgical complications, there were no neurovascular complications in neither group. The occurrence of proximal junctional kyphosis (PJK) [14] was 13% and 6% for the 51TLIF and OLIF51 groups, respectively; however, there were no statistical differences between the two groups. The instrumentation failures included set screw dislodge or rod fracture, with or without revision surgeries. The occurrence of instrumentation failure was 5% and 8% for the 51TLIF and OLIF51 groups, respectively, which was statistically equivalent (NS). In the OLIF51 group, we experienced a major intraoperative complication of pulmonary fat embolism requiring emergency extracorporeal membrane oxygenation (ECMO) treatment in the intensive care unit. The patient successfully recovered and completed the two-stage surgery. 

### 3.1. Case Presentations

#### 3.1.1. Case 1: 78 Years Old, Female, Degenerative Lumbar Scoliosis

**Figure 5:**
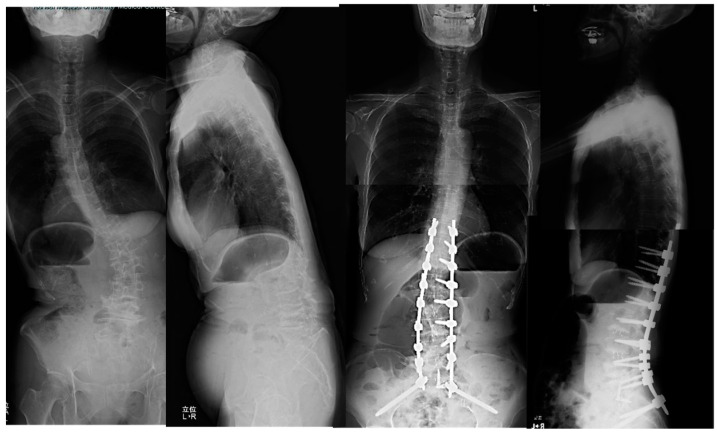
Seventy-eight-year-old female, degenerative lumbar scoliosis. The successful cMIS with OLIF51 was performed with a total blood loss of 294 mL.

The patient complained of severe low back pain, difficulty in standing, and pain and numbness in lower extremities preoperatively. She received cMIS employing OLIF51 with an intraoperative estimated blood loss of 294 mL and operation time of 418 min in total. The lumbar lordosis increased 4 to 54 degrees postoperatively and the PI-LL mismatch became 0 degrees postoperatively (Figure 5). 

#### 3.1.2. Case 2: 68 Years Old, Female, Adult Scoliosis

The patient suffered from severe low back pain and deformity for several years.

**Figure 6:**
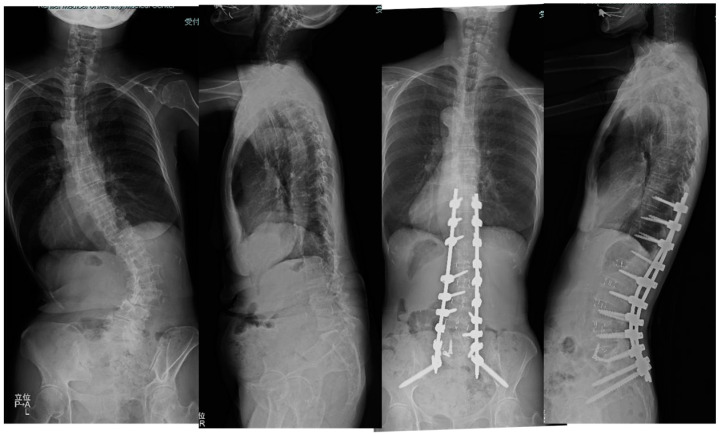
Sixty-eight-year-old female, adult scoliosis. The successful cMIS with OLIF51 was performed with a total operation time of 374 min and estimated blood loss of 594 mL. The preoperative Cobb angle of 63 degree was corrected to 7 degrees at follow-up. The preoperative PI-LL of 54 degrees was corrected to 2 degrees at follow-up.

She received cMIS employing OLIF51 with an intraoperative estimated blood loss of 594 mL and operation time of 374 min in total. The preoperative Cobb angle of 63 degree was corrected to 7 degrees at follow-up. The preoperative PI-LL of 54 degrees was corrected to 2 degrees at follow-up (Figure 6). 

LL was corrected to 54 degrees from 4 degrees and the PI-LL mismatch became 0 at follow-up.

## 4. Discussion

Historically, the management of adult spinal deformities has been a challenging for spine surgeons [15]. Adult spine deformity surgery carries a greater risk for several complications. The exclusive posterior or combined anterior–posterior surgery has been advocated in conjunction with posterior column or three-column osteotomy for the effective release for rigid spines [1,15]. However, these surgical modalities involve excessive estimated blood loss, longer operation time, and higher rate of postoperative complications [1,7]. 

In recent years, multiple lateral interbody fusions (LIFs) have been widely used with posterior open instrumentation (hybrid) or cMIS (all PPS) procedure for ASD [1,16,17]. Bae et al. compared three surgical strategies of an exclusive posterior approach, hybrid, and anterior fusion with a posterior approach for ASD [16]. Although satisfactory radiologic outcomes were achieved in the three groups, the hybrid group demonstrated lower PJK and mechanical complications, lower ODI scores, and higher SRS-22 scores [16]. Haque et al. examined 234 ASD patients and compared three modalities of open, hybrid, and cMIS surgeries. The cMIS group demonstrated a significantly smaller blood loss, while a larger PI-LL correction was achieved in the hybrid group. The lower complication rate was found for the cMIS and hybrid groups compared to the open group [18]. Park et al. compared two surgical strategies, i.e., hybrid and cMIS, in 105 ASD patients [19]. Although there was no difference in terms of radiologic parameters, ODI, and VAS scores, the complication rate was significantly lower in the cMIS than the hybrid group [19]. 

The OLIF51 procedure is a minimally invasive ALIF performed in the lateral decubitus position [8]. Using a small oblique incision medial to ASIS, the L5/S1 intervertebral disc is exposed via a retroperitoneal approach following the release and retraction of bilateral common iliac vessels. After an extensive disc excision, a bone graft and cage insertion with a high lordotic cage (up to eighteen degrees) can be placed with integrated screw fixation. Several papers have advocated for the advantage of OLIF51 over L5/S1 TLIF in single-level to multilevel reconstruction surgeries [11,12,20,21]. Mun et al. compared OLIF51 and L5/S1 TLIF in 148 patients for single-level fusion surgery [20]. They demonstrated that OLIF51 achieved a significantly higher disc angle than L5/S1 TLIF (22.6 vs. 12.3 degree), as well as a smaller cage subsidence rate of 16.2% (vs. 25.3%). Our previous study comparing single-level OLIF51 versus TLIF demonstrated that OLIF51 showed a higher fusion rate of 97% (vs. 92%), better low back function in the JOABPEQ effectiveness rate, and higher segmental lordosis at 17 degrees (vs. 12 degrees) [11]. For these reasons, the OLIF51 procedure has been applied to ASD surgery by several surgeons in the world [21,22,23]. 

We started employing the use of OLIF25 in 2012 for ASD, and applied it to L1–5 levels in conjunction with L5/S1 TLIF. Our posterior approaches have varied with the deformity severity for the hybrid, cMIS, and posterior approaches with osteotomy. Although the deformity correction and lower invasiveness of surgery were achieved successfully according to this surgical strategy, mechanical complications, including rod fracture, pseudarthrosis, and PJK, were still observed [24,25,26]. Since 2015, we have been employing OLIF51 combined with cMIS instead of 51TLIF. This study aimed to clarify the clinical and radiologic effects of the OLIF51 procedure with cMIS when compared to 51TLIF with cMIS. 

In ASD surgery, several studies have compared ALIF to TLIF at L5/S1 [27,28,29]. Dorward et al. conducted a matched cohort analysis of 42 patients in each group of ALIF and TLIF, demonstrating that ALIF resulted in significantly better segmental lordosis and less intraoperative blood loss over TLIF [27]. Singh et al. retrospectively investigated the pseudarthrosis rates of 49 patients of TLIF versus 51 patients of ALIF, with a two-year follow-up [28]. The results demonstrated that the clinical pseudarthrosis rate was significantly higher in TLIF (12/49) than in ALIF (1/51). The multivariate analysis revealed that the pseudarthrosis risk was 4.86 times higher in TLIF [28]. Adogwa et al. examined rod fracture rates between TLIF and ALIF in long-segment adult deformity surgery [29]. The result demonstrated that the incidence of a bilateral rod fracture was significantly higher in TLIF compared to ALIF (2.2% vs. 0.70%).

In terms of the comparison between OLIF51 and TLIF, there have been a few studies reported for ASD surgeries [21,22,23]. Park et al. reported a series of 23 patients, comprising 13 patients of OLIF51 and 10 patients of TLIF [23]. Although the operation time was statistically equivalent, the estimated total blood loss was significantly lower in the OLIF51 group (260 vs. 423 mL). Radiographically, the larger segmental angle (18.4 vs. 6.9 deg.), smaller PI-LL mismatch (3.6 vs. 7.5 deg.), and larger LL (55.5 vs. 46.9 deg.) and LLL (31.1 vs. 22.3 deg.) were demonstrated in the OLIF51 compared to the TLIF group [23]. Tanaka et al. clinically and radiographically compared 54 ASD patients, with 13 patients comprising OLIF51 and 41 patients TLIF [21]. They demonstrated shorter operation time (356 vs. 492 min) in OLIF51; however, no significant reduction in total estimated blood loss was identified (1016 vs. 1252 mL). Radiographically, a larger segmental angle (21.4 vs. 12.1) and higher disc height (11.3 vs. 9.4 mm) were statistically demonstrated in OLIF51; however, the OLIF51 group inversely showed a larger SVA and PI-LL mismatch than the TLIF group [21]. The superiority of LL and LLL creation in OLIF51 was not demonstrated in this study. In both studies, no statistical difference in the PJK and rod breakage rate, and clinical outcomes of ODI and VAS were demonstrated between OLIF51 and TLIF [21,23]. 

The present study demonstrated that OLIF51 resulted in a significantly shorter operation time and less estimated blood loss compared to TLIF. We speculated two reasons for this: first, TLIF required facet resections and second, the OLIF51 procedure led to better lordotic correction, enabling an easier and faster posterior correction procedure and less invasiveness of the surgery. Radiographically, OLIF51 provided higher segmental lordosis at L5/S1, thereby leading to better spinopelvic alignment and LLL over 70% lumbar lordosis. We found a significant difference in the L5 coronal tilt between the OLIF51 and TLIF groups. This meant that the OLIF51 procedure achieved a three-dimensional correction at the L5/S1 segment in both coronal and sagittal planes. In previous reports, the fractional curve correction in ASD was studied in terms of the use of ALIF [30,31]. Geddes et al. compared the fractional curve correction of ASD between 31 cases of L5/S1 ALIF and posterior spinal fusion (PSF) versus 28 cases of PSF alone. The results demonstrated a significantly greater coronal correction in the ALIF and PSF group compared to PSF alone. Buell et al. conducted a multicenter study comparing the fractional curve correction between L5/S1 ALIF versus TLIF in 106 patients of long-segment fusion for ASD [31]. The results demonstrated a comparable fractional curve correction between ALIF (66.7%) and TLIF (64.8%). In reviewing the previous studies employing OLIF51, this study was the first to demonstrate the superiority of OLIF51 in the coronal plane correction of ASD in addition to the sagittal plane. 

There were several limitations in this study. First, the patient sample size for the OLIF51 group was much smaller than that for the TLIF group. As this may influence the generalizability coming from our data, future research will employ a higher number of cases, as well as the use of a propensity score matching design to precisely compare the difference between the two surgical groups. Second, the follow-up periods differed, with 16 to 84 months for 51TLIF, and 13 to 42 months for the OLIF51 group. Although the bony fusion at the segments can be evaluated mostly at the 12 month period, this difference may influence the occurrence of a rod fracture and other instrumentation failures. Also, the quality-of-life parameters and VAS scores may be influenced. Third, we utilized an 18-degree cage (Capstone, Medtronics, Memphis, TN, USA) combined with autograft bone for L5/S1 TLIF. Although sixteen degrees of segmental lordosis was obtained at the L5/S1 segment, the use of the recently developed hyperlordotic cage or expandable cage may change the lordotic creation as well as coronal plane correction, even during the TLIF procedure.

## 5. Conclusions

The introduction of OLIF51 for adult spine deformity surgery led to a decrease in operation time and estimated blood loss, as well as improvement in sagittal and coronal correction compared to TLIF. The circumferential MIS correction and fusion with OLIF51 serve as an effective surgical modality which can be applied to many cases of adult spinal deformity.

## Figures and Tables

**Table 1:** Patient background demographics.

	OLIF51	TLIF
Patient number	35	82
Age at Surgery	75.0 (54–85)	75.0 (64–86)
Gender (Male: Female)	5: 30	14: 68
Weight (kg)	48.3 (32–68)	49.5 (32–72)
Height (m)	1.50(1.8–1.64)	1.51 (1.30–1.69)
BMI (%)	21.6 (13.6–30.0)	21.7 (15.8–30.2)
Scoliosis Cobb Angle (deg)	29.0 (0–72)	25.1 (3.8–45.2)
CVA (mm)	33.9 (0–112.7)	51.2 (19.7–123.5)
SVA (mm)	149.0 (0–691.0)	148.0 (10.5–349.2)
PT (deg)	47.8 (22.7–69.3)	52.2 (38.9–68.7)
LL (deg)	9.5 (−38.0–50.1)	7.6 (−29.3–37.0)
PI-LL (deg)	39.4 (0–77.6)	44.6 (4.3–91.5)
Seg. Lordosis at L5/S1 (deg)	11.3 (−3.0–29.7)	10.5 (−1.1–22.2)
Fixed Segments	7.7 (3–10)	8.1 (4–12)
OLIF Segments	4.6 (1–5)	3.9 (2–6)

Remarks: The average number and range are depicted in each category. There was no statistical difference for all categories between OLIF51 and MIS-TLIF. BMI: body mass index, CVA: coronal vertebral axis, SVA: sagittal vertebral axis, PT: pelvic tilt, LL: lumbar lordosis, PI-LL: pelvic tilt–lumbar lordosis.

**Table 2:** Summarized radiologic results for the two groups.

	OLIF51	TLIF	Significance
Preop. Cobb angle (deg)	29.0 (0–72)	25.1 (3.8–45.2)	NS
Follow-up Cobb angle (deg)	10.0 (0–28)	9.5 (1.4–23.3)	NS
Preop. thoracic kyphosis (deg)	23.8 (2.1–73.0)	20.9 (1.1–77.7)	NS
Follow-up thoracic kyphosis (deg)	38.3 (18.1–59.0)	36.0 (13.6–57.9)	NS
Preop. CVA (mm)	33.9 (0–112.7)	51.2 (19.7–123.5)	NS
Follow-up CVA (mm)	17.0 (0–58)	29.2 (0–53.1)	NS
Preop. SVA (mm)	149.0 (0–691)	148.0(10.5349.2)	NS
Follow-up SVA (mm)	26.9 (−35.0–99.0)	33.7 (−33.1–112)	NS
Preop. SS (deg)	17.0 (−28–44)	21.4 (11.6–33.4)	NS
Follow-up SS (deg)	30.0 (11–50.5)	28.3 (15–35.4)	NS
Preop. PT (deg)	31.2 (14–54.0)	30.9 (18.3–48.1)	NS
Follow-up PT (deg)	19.0 (−3.0–35.0)	16.7 (4.9–36.0)	NS
Preop. LL (deg)	9.5 (−38.0–50.1)	7.6 (−29.3–37.0)	NS
Follow-up LL (deg)	46.9 (22.0–65.0)	45.4 (33.2–58.9)	NS
Preop. LLL (deg)	20.2 (−26.7–47.2)	18.1 (−10.4–34.8)	NS
Follow-up LLL (deg)	34.9 (13.0–51.7)	28.8 (21.0–34.0)	*p* < 0.01
Preop. PI-LL (deg)	39.4 (0–77.6)	44.6 (4.3–91.5)	NS
Follow-up PI-LL (deg)	2.5 (−23.7–24.0)	6.9 (−9.6–33.6)	*p* < 0.05
Preop. L5/S1 segmental lordosis (deg)	11.3 (−3.0–29.7)	10.5 (−1.1–22.2)	NS
Follow-up L5/S1 segmental angle	19.8 (5.6–44.1)	16.0 (4.5–21.0)	*p* < 0.01
Preop. L5 coronal tilt (deg)	6.0 (0–230)	5.4 (0.6–14.5)	NS
Follow-up L5 coronal tilt (deg)	2.5 (0–8.0)	4.3 (0–10.5)	*p* < 0.01

Remarks: The average number and range are depicted in each category. Statistical difference is shown in the right column. NS: no statistical difference, Preop.: preoperative, CVA: coronal vertebral axis, SVA: sagittal vertebral axis, SS: sacral slope, PT: pelvic tilt, LL: lumbar lordosis, LLL: lower LL, PI: pelvic tilt.

**Table 3:** Visual analogue scale for the two groups.

	OLIF51	TLIF	Significance
Preop. LBP	58 (0–100)	63.6 (30–80)	NS
Follow-up LBP	16.4 (0–50)	21.0 (0–70)	NS
Preop. LE pain	58.3 (0–100)	52.7 (20–70)	NS
Follow-up LE pain	17.3 (0–80)	26.0 (0–90)	NS
Preop. LE numbness	43.4 (0–100)	51.8 (20–70)	NS
Follow-up LE numbness	17.3 (0–60)	11.0 (0–30)	NS

## Data Availability

The data presented in this study are available in the article.
